# Virtual linear measurement system for accurate quantification of medical images

**DOI:** 10.1049/htl.2019.0074

**Published:** 2019-12-02

**Authors:** Gavin Wheeler, Shujie Deng, Kuberan Pushparajah, Julia A. Schnabel, John M. Simpson, Alberto Gomez

**Affiliations:** 1 School of Biomedical Engineering & Imaging Sciences, King's College London, London, UK; 2 Department of Congenital Heart Disease, Evelina London Children's Hospital, London, UK

**Keywords:** rendering (computer graphics), echocardiography, image reconstruction, medical image processing, virtual reality, virtual linear measurement system, virtual reality, volumetric medical images, clinical image analysis tool, VR applications, Unity-based system, linear measurements, 3D echocardiographic images, image analysis packages, 3D measurement tools, 3D volume rendering measurement tools

## Abstract

Virtual reality (VR) has the potential to aid in the understanding of complex volumetric medical images, by providing an immersive and intuitive experience accessible to both experts and non-imaging specialists. A key feature of any clinical image analysis tool is measurement of clinically relevant anatomical structures. However, this feature has been largely neglected in VR applications. The authors propose a Unity-based system to carry out linear measurements on three-dimensional (3D), purposefully designed for the measurement of 3D echocardiographic images. The proposed system is compared to commercially available, widely used image analysis packages that feature both 2D (multi-planar reconstruction) and 3D (volume rendering) measurement tools. The results indicate that the proposed system provides statistically equivalent measurements compared to the reference 2D system, while being more accurate than the commercial 3D system.

## 1 Introduction

Over the last 50 years, medical imaging and particularly echocardiography have experienced a tremendous revolution, going from initially acquiring one-dimensional (1D) scan lines, then 2D dynamic images, and to current high resolution, high frame-rate 3D real-time images of the heart. However, the fundamental means of display has been limited to 2D screens, first for 2D images and later to flat projections of 3D images. The advent of 3D display technology, including holography or virtual and augmented reality (VR/AR), to cite a few examples, has recently enabled development of immersive applications to visualise and interact with 3D medical data [1]. Most VR medical software allows visualisation of and interaction with 3D surface models that can be extracted from medical images through segmentation [2, 3], or direct rendering of 3D medical images in VR [4, 5], often for advanced surgery planning [6]. However, most surgery planning software requires the ability to measure the anatomy, and this feature is largely lacking in existing VR medical applications.

In this Letter, we describe a system for making 3D measurements in VR for medical image applications, and specifically for 3D echocardiographic images. The contribution of this Letter is twofold: first, we describe the requirements associated to 3D measurements in VR, and propose a Unity solution and implementation; and second, we evaluate the proposed measurement system by comparing it to measurements on the same datasets carried out by clinical experts on commercial clinical systems.

## 2 Related work

Echocardiography is the modality of choice when planning most cardiac procedures, and is sometimes complemented with other imaging modalities such as magnetic resonance imaging and computed tomography (CT). Commercial software is available for cardiac procedure planning, and representative examples of widely used programs for echocardiography are Philips QLAB and Tomtec Cardioview. Amongst its functionality, Philip's QLAB offers basic measurement tools including distance of a straight line on multi-planar reconstruction (MPR). Measurement of 3D structures using MPR requires advanced skills to navigate through complex 3D anatomy, and 2D slices are unable to capture non-planar structures. As a solution to this, Tomtec additionally offers measurement on volume rendering including linear distances in 3D. Unfortunately, the 3D rendered volume is projected into a flat screen, limiting 3D perception, and each measurement is dependent on the specific orthographic view it was made in.

Recently, commercial VR tools for 3D medical images have been made available (DICOM VR [www.dicomvr.com.], The Body VR – Anatomy Viewer [www.thebodyvr.com.]), some with VR measurement capabilities (Medical Holodeck [www.medicalholodeck.com.]) although no evidence on its suitability for clinical use is available. Open source initiatives (VR 3D Slicer [7]) and generic frameworks to integrate medical imaging libraries with VR toolkits [5] have been recently proposed. The quality of the volume rendering from this solution was assessed by clinicians and found to be clinically acceptable [8], additionally the VR system was found to be comfortable, and overall was preferred to standard tools.

Making measurements in volume data has long been a subject of investigation. For instance, Preim *et al.* [9] and Reitinger *et al.* [10] both describe desirable properties of manual measurement systems in 3D, the latter implemented in AR. Examples of such properties are that measurement end points should be clearly visible and adjustable, the measurement value is always visible to the user and measurements respond to scaling of the volume.

In spite of the vast literature on VR/AR medical applications, published work on evaluation of 3D measurements in VR is scarce. Reitinger *et al.* found improved accuracy and speed of their Virtual Liver Surgery Planning System (displayed on a CAVE-like system with shutter glasses) when compared to Osirix [11]. However, the VR data were displayed as a surface rendering based on a segmentation, as opposed to volume rendering of the original CT data in Osirix, and the participants were neither imaging nor Osirix experts. Verwoerd-Dikkeboom *et al.* compared 4D View (GE) and the Barco I-Space CAVE-like system performance measuring foetal biometric parameters [12]. They showed that both systems made comparable, reliable measurements. They discussed potential interaction advantages of VR over 2D views, but did not evaluate them.

A mid-air hand gesture controlled measurement system implemented using the Leap Motion controller with display on a large 2D screen was evaluated by Saalfeld *et al.* [13]. Compared to mouse and keyboard control, gesture control was found to be slower, less accurate and more tiring. However, for the target interventional application, maintaining a sterile environment makes mid-air gesture control desirable.

Automatic measurement systems based on segmenting the image have also been investigated, for instance [9, 14]. However, for our application segmenting the image is undesirable as we want to work directly with the volume data since changes in gain and contrast can alter apparent boundaries making segmentation challenging.

In this Letter, we report a Unity implementation of a linear measurement system for 3D images in VR, and carry out a quantitative and qualitative comparison with respect to commercially available software using data from cardiac patients.

## 3 Methods

We first describe the generic requirements for a 3D linear measurement system in VR, and then describe a solution that satisfies these, whilst maintaining interactivity and ensuring the integrity of the measurements. A linear measurement tool for VR should allow the user to draw a line in 3D and provide its length, as follows:

- The user must have means of defining the measurement by its start and end points, in the VR space.
- The visual representation of the measurement should move and scale as the measured object is moved and scaled.
- The measured distance should remain invariant to such transformations.
- The measurement should be editable (start, end, label, whole measurement).
- The visual representation of the measurement should be integrated, but not obscured by, and not obscure, the data being measured.

Additionally, for convenience we tailor our solution to leverage the Unity development environment, and use VTK for volume rendering following the plug-in method described in [5]. This allows the use of a wide range of VR hardware, particularly the HTC Vive (which we use in our experiments). This method embeds VTK imaging props (for instance, the volume rendering, 2D MPR slices) into proxy Unity game objects that do not have their own render mesh. The Unity game objects and VTK props are linked through the plug-in system, so that when the user interacts with the Unity proxy object, that interaction is reflected in the VTK object.

**Fig. 1:**
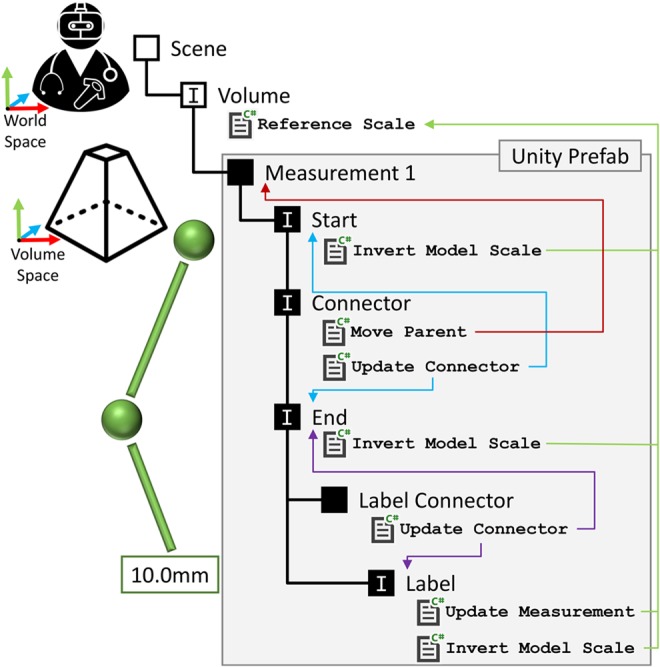
Hierarchical structure of the measurement prefab, as implemented in Unity. The measurement object has five child objects, as illustrated. Objects marked with ‘I’ have physics interactors. Blue and purple arrows indicate the linking of the connector lines to the start point, end point and label. Green arrows indicate the Unity scripts governing the scale of the objects. The red arrow indicates the redirection of editing (translate, rotate) from the connector to the measurement parent. Shapes, colours and label text are a representative example

We propose a hierarchical structure for a measurement representation, implemented as Unity prefab, and illustrated in Fig. 1. This hierarchy enables a compact representation of a measurement consisting of start and end points, a line connecting them, a label showing the measured distance and a second line linking the label to the end point. In the following sections, we describe the specific mechanisms that allow this structure to fulfil the requirements above.

### 3.1 Defining the start and end points

By default Unity's physics engine has a world space defined in metres, a parallel of the real world. The SteamVR asset for Unity tracks the VR headset and controllers in the real world, and directly transfers their positions and orientations to Unity's world space. Thus, a controller may directly define a 3D point in the Unity world space. In our implementation, utilising the HTC Vive controller, a measurement marker is attached to the representation of the controller in the VR space. The user places this marker at the starting point for the measurement, and pulls the controller's trigger. This creates the measurement object (replacing the marker), fixes the measurement start point transform in the virtual space and switches to moving the measurement end point which is now attached to the controller. The user then defines the end point by moving the end point marker to the desired location and releasing the trigger.

### 3.2 Tracking the movement and scale of the volume

The visual representation of the measurement should translate, rotate and scale with the volume, so that the measurement remains at the same anatomical position. The Unity scene graph is used to manage this – the measurement is a child of the volume in the scene graph, so the measurement will track the volume's position, rotation and scale.

### 3.3 Distance calculation and scale invariance of the measurement

This is perhaps the single most important aspect – if the measurements are not reliable and in real-world units the tool will not be of clinical value. Unity's world coordinate system, defined in metres, does not match common medical image units (typically mm, usually defined in the image header). In our case, we use the VTK plugin to scale the volume data it loads to match the Unity world scale of metres [5]. When the volume data are visualised, the Euclidean distance between the measurement start and end points can be trivially computed either in Unity's world space (defined in Unity's scene), or in the local volume space (defined by the axes of the volume cube), because their scales match.

We implement this through a Unity C# script, which has references to the measurement start and end point Unity game objects. It calculates the distance between these and updates the text in a Unity UI.Text render object used to display the measurement distance to the user. This calculation is performed in the Update Measurement script's Update() method – so the measurement text is interactive, updated every render frame.

However, the user will often scale (zoom) the rendered volume for improved visualisation – which may affect the measured distance. If the measurement is computed in volume space, by using the local positions of the start and end point transforms in the distance calculation, the volume's scale will not affect the measured distance – since the local positions of the measurement start and end points do not change. However, in our implementation this method not was pursued because it is incompatible with the editing of the measurement points – editing the measurement makes the start or end point a child of the controller, so that point's local position is no longer in volume space.

Instead we calculate the measurement distance using the Unity world space. Due to this, the distance between the start and end points calculated in world space must be inversely scaled by the volume visualisation scale. To implement this, we first added a Unity C# script to the volume visualisation game object – Reference Scale. This script simply labels that object as having the ‘reference scale’. Secondly, we modify the distance calculation script to search for the object with this script, and then inversely scale the distance calculation by the scale of the reference object.

### 3.4 Measurement editing

Editing the measurements, for instance moving the start or end point, was implemented using the triggers in Unity's physics system. A small, trigger physics box is attached to the controller game object. Non-trigger physics boxes are attached to the elements in the scene which may be picked up and moved, for instance start and end points, measurement label. When the controller physics box intersects with one of these, the user may ‘pick-up’ the scene object up by pulling the controller trigger. This is achieved by re-parenting the scene object to the controller game object – as a child of the controller the scene object will then naturally track the controller's motion. When the user later releases the trigger the original parent of the scene object is restored.

### 3.5 Visual representation

**Fig. 2:**
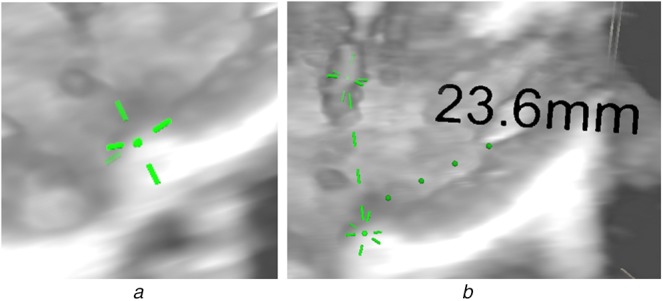
Measurement display *a* Visualisation of the end point with sphere and spokes *b* Display of the whole measurement, including start point, end point, label and connectors, in a volume rendering

The markers at the ends of the measurement need to be small enough to mark a point, large enough to be clearly visible, but not so bold as to obscure the anatomy of interest. Based on existing 2D tools and on end-user feedback, our proposed end point model has a central sphere (providing a definite, small point) and a spoke either side of this sphere along each of the *X*, *Y*, *Z* axes – making the marker clearly visible while minimising obstruction of the anatomy. An example of this model is shown in Fig. 2*a*. A final benefit of the spokes is that as a measurement point is placed on the surface of a structure in the volume, the spokes inside the structure become obscured – helping placement.

The start and end points are connected by a dashed line connector, updated each frame by the Update Connector script (Fig. 1). This connector is represented by a series of thin cylinder prefabs, whose location and orientation is updated each frame to connect the start and end points. As the distance between the start and end points increases and decreases cylinders are created and destroyed, to maintain regular spacing. Additionally, the connector has a physics box which is translated, rotated and scaled to maintain consistency with the dashed line. The scaling of this only takes place along one axis – so the physics box may get longer, but not thicker. This physics box allows the connector to be detected by the editing tool, and so used to pick up and move the whole measurement. The Move Parent script redirects the editing tool to move the measurement game object, so moving every element in the prefab, rather than just the connector. An example of measurement is shown in Fig. 2*b*.

Additionally, while the length of the visual representation of the measurement should scale with the volume, it should not change point marker size or connector line thickness – else it would unnecessarily obscure the volume when zooming in, or become invisible when zooming out. The Invert Model Scale script attached to the start point, end point and label objects invert the scale of their respective render meshes when compared to the ‘reference scale’. So, as the user zooms into the volume, the measurement of their world size remains constant, and so they do not obscure the user's view.

## 4 Materials

We compare the accuracy and usability of three systems for making linear measurements on 3D echocardiography data: Philips’ QLAB making measurements on MPR (which we use as reference method), Tomtec Echoview making measurements on volume rendering, and our proposed VR measurement system making measurements on volume rendering. A QLAB measurement has a precision of 0.1 mm, a Tomtec measurement has a precision of 1 mm and a VR measurement has a precision of 0.1 mm. QLAB and Tomtec both loaded anonymised DICOM data. For use in our VR application, the test data were converted to Cartesian DICOM using Philips’ QLAB plug-in, which in turn were converted to a VTK compatible format to be read by our application using a Python script. The QLAB reference clinical imaging platform ran Philips QLAB 10.8, and was used either on a standard hospital workstation or on a Dell Precision 5510 laptop with a 15″ 1920 × 1080 resolution screen. Tomtec was run on the same systems. Our proposed VR system ran on a Dell Alienware desktop with a Core i7-8700 3.2 GHz processor, 32 GB RAM, and an Nvidia GTX1080Ti graphics card with 11 GB RAM, connected to an HTC Vive headset and two controllers.

Two types of datasets were used: a 3D image of an ultrasound calibration phantom (Gammex 403GS LE) with cylindrical inserts of known sizes (4, 6 and 10 mm in diameter); and four datasets from congenital cardiac patients. All data were acquired using a Philips EPIQ 7 using a X5-1 matrix array transduce. All data had been acquired for clinical reasons. Ethical approval for research measurement of anonymised data has been approved.

Five cardiologists, with the following profiles, participated in a user study: 3 imaging cardiologists/2 physiologists; 4 senior (5 + years of experience)/1 junior (<5 years experience); all use QLAB almost daily, and Tomtec at least once a month or once a week, though none used it on a daily basis; 3 use VR ‘nearly every month’/2 only rarely; 4 used basic measurement tools almost every day/1 weekly; 1 used advanced measurement tools daily/3 at least weekly/1 monthly.

## 5 Experiments

We run a user study to evaluate the proposed measurement tool against clinical, commercially available tools (QLAB and Tomtec). All participants were trained before the experiments so they felt confident of all the interactions needed in the measurement tasks. All participants made the measurements described in Table 1. Participants were free to explore the data, and asked to make the measurements as accurately as possible. They were free to edit the measurements until they were satisfied. They were free to alter the view and the gain and contrast of the rendering transfer function. The measurements to be made and the cardiac phase to use (specified by name and frame number) were described to the participants through the experiment. Following their use of all of the tools all participants answered a questionnaire.

**Table 1:** Summary of measurements used for evaluation: three measurements on the calibration phantom, and five measurements on real CHD echo datasets

Dataset type	Cardiac view/window	Cardiac phase	Measurements
US calibration phantom (Gammex 403GS LE)	N/A	N/A	vertical and horizontal diameter of the low scatter cysts (4, 6, 10 mm)
US patient data	parasternal long axis view	systole	aortic valve hingepoint
			left atrial dimension
	short axis view	diastolic	left ventricular end diastolic dimension
		end systolic MV closed	left ventricular end systolic dimension
			aortic end systolic dimension

To avoid biasing the participants, the measurement distance labels were covered from view in QLAB and Tomtec. Our VR system was modified so that the measurement distance was hidden from the participant, but visible to the experiment observer. Results were recorded during each set of measurements, and then transferred to Microsoft Excel for analysis.

The measurements on phantom data were used to test for accuracy and precision of the measurement systems, compared to the known distance. Patient data was used to compare performance between systems for a clinically relevant task: measuring relevant anatomical features. These standard clinical measurements were chosen as they are all important for assessing heart function in congenital heart disease (CHD). In total, each participant carried out 26 measurements (5 measurements on each of the 4 patient datasets plus 6 measurements on the phantom dataset). We used these data to compute the similarity between the measurements using QLAB (reference) and both Tomtec and VR measurements, using a *t*-test; and intra-user variance for the three tools.

After the measurement session, participants completed a questionnaire, with the following questions (and valid answers): (i) confidence on measurement with each tool (score 1 to 4); (ii) what makes you confident or lack confidence in your measurements, with each tool (free text); (iii) what new functionality in the VR system would help you make confident measurements (free text), (iv) what is the perceived influence of brightness, contrast, size of structure and nature of data on measurement accuracy (it affects or not, and how). Participants were also asked open ended questions to record perceived advantages and disadvantages of the proposed system.

## 6 Results

### 6.1 Precision, accuracy and variability of measurements

Results of measurements on phantom data (which reflect precision and accuracy) are provided in Table 2, showing the ground truth dimensions (left column) and the corresponding average and standard deviation measurements (over participants). One Tomtec measurement has zero standard deviation because all participants measured the same distance (a corollary of Tomtec's 1 mm measurement display precision). The best precision (smallest standard deviation) is achieved with QLAB, while the best accuracy (smallest mean error) is obtained with the proposed VR system.

**Table 2:** Measurement of the diameter of six cylindrical inserts in an ultrasound image of a calibration phantom Gammex 403GS LE. All diameters were measured both horizontally (H) and vertically (V)

Phantom, mm		QLAB, mm		Tomtec, mm		VR, mm	
Size	H/V	Mean	SD	Mean	SD	Mean	SD
4.0	V	3.4	**0.508**	2.8	0.837	**4.4**	0.449
4.0	H	2.9	**0.397**	3.2	0.447	**3.8**	0.778
6.0	V	5.3	**0.219**	4.2	0.837	**5.9**	0.770
6.0	H	4.9	**0.241**	5.0	**0.000**	**6.3**	1.113
10.0	V	**10.3**	**0.652**	9.0	0.707	10.4	0.920
10.0	H	**9.6**	**0.205**	10.4	0.548	11.7	1.882

Mean result closest to the reference and the smallest standard deviations are shown in bold.

**Fig. 3:**
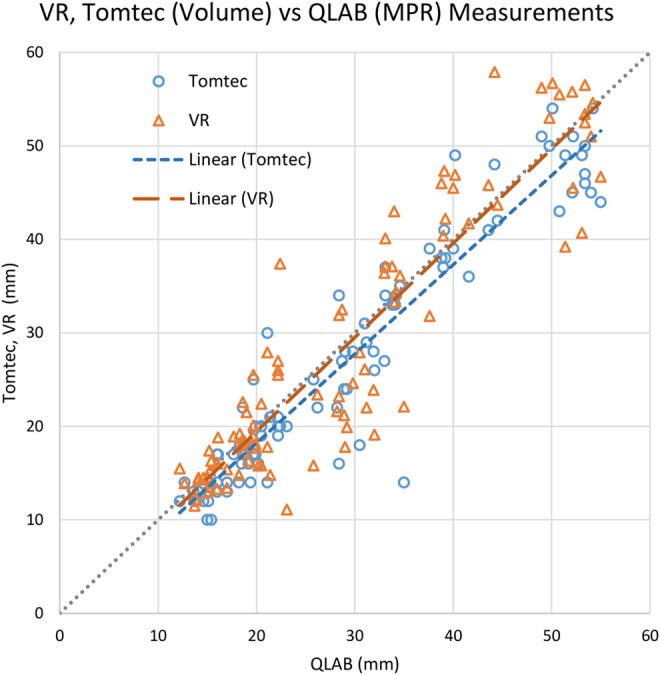
Comparison of VR and Tomtec volume measurements referred to QLAB's MPR measurements, carried out on patient data. Markers represent individual measurements, and the dashed lines indicate linear regressions of the data points

Fig. 3 illustrates the measurement consistency between QLAB (our baseline, on the horizontal axis) and both Tomtec and the proposed VR system (3D measurement tools, on the vertical axis). In all cases, a high correlation is shown. For Tomtec, 17 out of 26 measurements were less than 2 mm different between the two tools. The mean difference is − 1.57 mm $ \pm $ 2.41 mm. Using our proposed VR system, 15 out of 26 measurements were under 2 mm apart between the two tools. The mean difference is 0.36 mm $ \pm $ 3.81 mm. These results are consistent with the phantom experiment, where our proposed VR system has higher accuracy but lower precision.

We conducted two paired-samples *t*-tests on the measurements, VR versus QLAB and Tomtec versus QLAB. Five observations were incorrectly recorded so their data were omitted. No significant difference was found between the measurements for VR: mean (*M*) = 28.386, standard deviation (SD) = 14.110 and QLAB (*M* = 28.882, SD = 12.881); *t*(94) = −0.871, *p* = 0.385. There was a significant difference between the measured distances with Tomtec (*M* = 26.684, SD = 12.951) and QLAB (*M* = 28.882, SD = 12.881), *t*(94) = −5.182, *p* < 0.001.

**Fig. 4:**
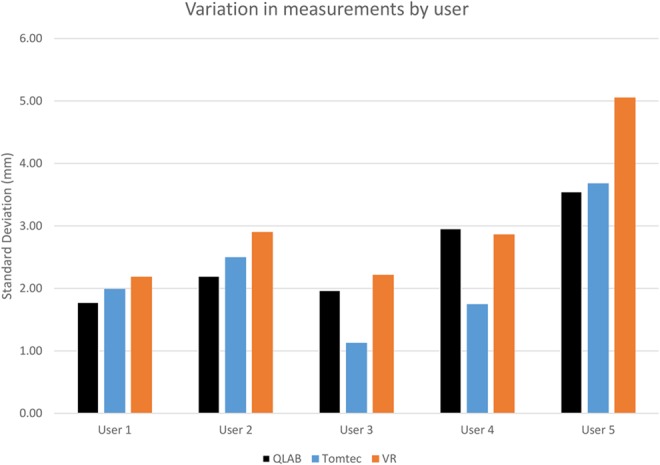
Variation in the difference in measurements made by QLAB, Tomtec and VR by user, comparing the difference between a user's measurement to the mean over all tools for that dataset + measurement + user combination

Fig. 4 shows the intra-user variability. For each tool we calculate the standard deviation of the differences between a user's measurement, to the mean of all that measurement made by the user using all tools.

Users 1 and 2 have the most consistent measurements between the three tools. They both have more than 5 years experience and use QLAB almost daily. User 1 uses Tomtec and VR monthly. They were ‘very confident’ of their QLAB measurements and ‘somewhat confident’ of their Tomtec and VR measurements. User 2 shows slightly greater variability. They use Tomtec monthly and VR rarely. They were ‘very confident’ of their QLAB measurements, ‘somewhat confident’ of their Tomtec measurements but ‘very much lacking confidence’ in their VR measurements.

User 3 has similar variability for QLAB and VR to User 1. However, they have lower variability for Tomtec, which they use more often – on a weekly basis, and have between 1 and 5 years experience. They were ‘very confident’ of their QLAB measurements and ‘somewhat confident’ of their Tomtec and VR measurements.

User 4 also has lower variability on Tomtec than the other tools, and like User 3 they use it on a weekly basis. They use QLAB daily, but have only used VR once or twice in the past, and have more than 5 years experience. User 4 was ‘somewhat confident’ of their measurements for all tools.

User 5 has the greatest variability of all participants. They use QLAB almost daily, and Tomtec and VR monthly, and have over 5 years clinical experience. Like User 4, they were ‘somewhat confident’ of their measurements for all tools.

### 6.2 User study

In terms of confidence in their measurements, all participants were ‘somewhat confident’ or ‘very confident’ with all tools, except for one participant reporting ‘very much lacking in confidence’ with the VR system. There was no correlation found between perceived confidence and achieved accuracy.

Open ended questions provided reasons for user confidence. Positive aspects of QLAB included ‘use frequently’, ‘the ability to be in orthogonal planes for the MPR’ and ‘the plane of crop is defined and edges not impacted by gain etc.’. However, negative responses included ‘hard to see the aortic ones in the far field, also edge definition’ and ‘they are very alignment specific and lacking the depth means you may over/underestimate significantly’.

For Tomtec users identified the following positive aspects: ‘I can see structures en face and am confident I am seeing the leading edge.’ and “I can ‘stand back’ from moving structures e.g. mitral valve”. Negatives user comments included ‘It is sometime blurry and the definition is not always very clear’, ‘edge detection to place marker – boundary sometimes less clear’ and ‘I don't use very often’.

For VR positive comments included: ‘knowing I am in the true long or short axis of a structure’ and “Intuitive to use. I can ‘stand back’ from structure being measured”. On the negative side users commented ‘The impact of image gain is a concern’, ‘Different tools and gain settings made me feel less than confident in placing the calipers’, ‘Sometimes it is a bit fuzzy and tone and shadow are harder to distinguish than in the other modalities’.

## 7 Discussion

We proposed a 3D VR system to carry out linear measurements on volumetric images, and demonstrated it on echocardiographic images of a calibration phantom and of cardiac patients. All measurements were carried out with Philips QLAB (our baseline), Tomtec (its 3D measurement system only) and our proposed VR platform. Evaluated by clinicians who use QLAB almost daily, we are setting our VR measurement system, with which they are so much less familiar, a stern test. The inclusion of Tomtec in the test gives us a reference point for a commercial tool in a similar situation.

Quantitatively, all systems are relatively close, with our system being the most accurate: for patient data, relative to QLAB, it is more accurate than Tomtec; and in absolute terms, using calibrated phantom data, it is the most accurate of the three systems. Indeed, the results obtained with Tomtec seem to be biased towards lower values. This could be related to the lack of true depth perception when looking at 3D structures on a flat screen, which our system solves. Although the precision results are not conclusive, they suggest that our system is the less precise. We hypothesise that this could be related to the lack of familiarity of the users with the system, but also with the fact that all measurements were drawn from protocols designed for MPR.

The user study showed a generally very positive attitude of clinical users towards VR imaging platforms. Intuitiveness and 3D perception were highlighted as positive points. Of slight surprise is that two users were only ‘somewhat confident’ in the measurements made using QLAB. As expected, confidence levels are lower for Tomtec than QLAB (which were similar to each other), as it is used less often. Looking into the reasons for this, some common themes stand out. First, MPR gives the viewer the ability to specify the viewing planes in a structured, systematic way which build confidence. Conversely, it also identified that unless the planes are correctly specified it is easy to underestimate or overestimate a measurement. Secondly, MPR is felt to be less affected by changes to gain, contrast and so on. As a matter of fact, participants seemed somewhat concerned about the impact that changes in gain and brightness may have on the perceived size of anatomical structures, defects and so on, on the rendered volume.

Many of these opinions can already be addressed. For instance, we have the ability to render MPR images in the VR scene, and it is technically feasible to link the measurements between the volume rendering and the MPR. Concerns with volume rendering can also be addressed, through better user control and more options, e.g. filtering and lighting. However, there is no such direct solution to these concerns – there is likely a need for a cycle of making changes and then reviewing them with clinicians. Knowing this will help us focus our efforts on the most fruitful aspects of development.

Overall, this study showed that a VR system can have measurement tools that are comparable to clinically used commercial tools, while providing further insight and understanding into complex 3D anatomy.

## 8 Funding and declaration of interests

This work was supported by the NIHR i4i funded 3D Heart project [II-LA-0716-20001]. This work was also supported by the Wellcome/EPSRC Centre for Medical Engineering [WT 203148/Z/16/Z]. The research was funded/supported by the National Institute for Health Research (NIHR) Biomedical Research Centre based at Guy's and St Thomas’ NHS Foundation Trust and King's College London and supported by the NIHR Clinical Research Facility (CRF) at Guy's and St Thomas’. The views expressed are those of the author(s) and not necessarily those of the NHS, the NIHR or the Department of Health.
